# RETRACTION: Meta‐Analysis of the Prognostic Value of Narcotrend Monitoring of Different Depths of Anesthesia and Different Bispectral Index (BIS) Values for Cognitive Dysfunction after Tumor Surgery in Elderly Patients

**DOI:** 10.1155/johe/9786279

**Published:** 2026-06-05

**Authors:** Journal of Healthcare Engineering

RETRACTION: N. Chen and J. Lu, “Meta‐Analysis of the Prognostic Value of Narcotrend Monitoring of Different Depths of Anesthesia and Different Bispectral Index (BIS) Values for Cognitive Dysfunction after Tumor Surgery in Elderly Patients,” *Journal of Healthcare Engineering* 2022, no. 1 (2022): 1–8, https://doi.org/10.1155/2022/8554188.

The above article, published online on 17 March 2022 in Wiley Online Library (https://wileyonlinelibrary.com), has been retracted by agreement between the journal Editor, David Hewson; and John Wiley & Sons Ltd.

The retraction has been agreed following an initial request from the corresponding author and subsequently identified concerns relating to overlap with a previously published article [1] where there are no common authors or affiliations.

The authors were informed of the retraction, but did not respond.
